# Supplementary material for: FasR Regulates Fatty Acid Biosynthesis and Is Essential for Virulence of Mycobacterium tuberculosis
Source: Front Microbiol. 2020 Oct 27;11:586285. doi: 10.3389/fmicb.2020.586285 (PMC7652896; doi:10.3389/fmicb.2020.586285)
Supplement: Supplementary file 1 [file Data_Sheet_1.pdf]

Supporting information

Table S1. Plasmids used in this study

Name	Description and features	References
pFR3	pET28a(+) with *fasR* His tag fusion gene, under the control of T7 promoter, Kan^R	(Mondino et al., 2013)
pJG1100	Suicide vector for mutant construction, Hyg^R, Kan^R, *sacB*	(Gomez and Bishai, 2000)
pFR35	Suicide vector for *Mtb*Δ*fasR* mutant construction derived from pJG1100, Hyg^R, Kan^R, *sacB*	This study
pGA44	Integrative vector at L5 *attB* site, carrying the TetR/Pip OFF expression system and Ptr promoter, Str^R/$Spect^R$ The *int* gene was removed in order to lock this plasmid at the integration site. Integration requires plasmid pGA80	(Kolly et al., 2014)
pGA80	pMV261-derived vector, carrying the L5 *int* gene for expression in *trans*, lacking the mycobacterial origin of replication (Δ*oriM*), Kan^R	(Kolly et al., 2014)
pFR33	pGA44-derived vector carrying *fasR* under the control of the Ptr promoter, Str^R/$Spect^R$	This study
pND255	L5-integrative vector for mutant construction, Hyg^R	(Kolly et al., 2014)

Hyg^R, hygromicin resistance; Kan^R, kanamycin resistance; Str^R/$Spect^R$, streptomycin/spectinomycin resistance

Table S2. Strains used in this study

Name	Description or Genotype	References
DH5α	*E. coli K12 F- ΔlacU169 (Φ80lacZΔM15) endA1 recA1 hsdR17 deoR supE44 thi-1λ- gyrA96 relA1*	(Hanahan, 1983)
*Mtb*H37Rv	*M. tuberculosis* wild type strain	(Cole et al., 1998)
*Mtb*Δ*fasR*	*Mtb*H37Rv background. Deletion of *fasR*	This study
*Mtb*Δ*fasR-cfasR*	*fasR* knockout complemented with *fasR* expressed *in trans*	This study

Table S3. Oligonucleotides used in this study

Name	Sequence (5'-3')
3208_1up	CTCGAGCCGGAATTCGTAGCC
3208_2down	CATATGGAGCGCTGCCGGACGATCTG
3208_3up	CATATGAAGCCGATTTCCAAGTCCGAC

3208_4down	ACTAGTTGCCACCGTGGTGACCGTCTC
Southern_1fasRTB	GAACACTTTGACGGTGCCTTG
Southern_2fasRTB	GGAATGACATTACTACCTGCG
L-*sigA*	AAACAGATCGGCAAGGTAGC
R-*sigA*	CTGGATCAGGTCGAGAAACG
L-*fasR*	TGAGCACGACTACCGACAAC
R-*fasR*	CTCGAAGATCAGCCGGTAAC
L-*fas*	GACATCCTGACCCGACTGAC
R-*fas*	TGAACTTCGTCGAGAGCTTG
fasRF	ATGAGCGATCTCGCCAAGACAG
fasRR	CTACGAGCGGGTAAGCGGGACG

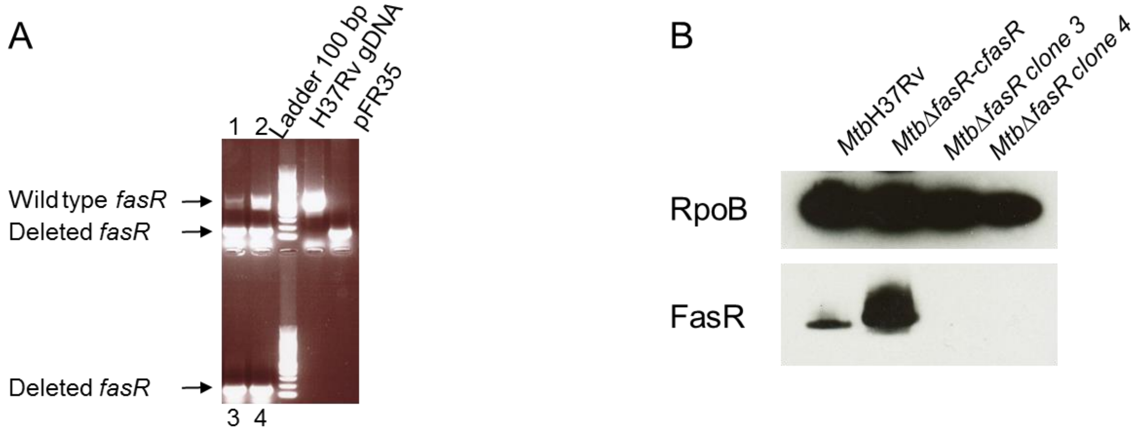

Figure S1. Analysis of the knockout *fasR* mutant (A) Colony PCR of *M. tuberculosis* transformants grown on Str, carrying the complementing *fasR* gene (clones 1 and 2) and of transformants grown on Hyg, carrying the empty pND255 vector (clones 3 and 4). Primers used for the amplification of *fasR* are listed in Table S3. The expected size of the PCR products is 687 bp for the *fasR* wild type allele and 150 bp for the mutant *fasR* allele. Two fragments were amplified from the Str-resistant clones, corresponding to the complementing *fasR* gene from the integrated plasmid and the deleted *fasR* gene at the *fasR* locus; on the other hand, only one fragment was amplified from the Hyg resistant clones (the partially deleted *fasR* gene) **(B)** Western Blot analysis of crude protein extracts prepared from cultures of wild type H37Rv strain (*Mtb*H37Rv), *fasR* conditional knockdown strain (*Mtb*Δ*fasR*-c*fasR*) and *fasR* KO strain (*Mtb*Δ*fasR* clones 3 and 4) grown at OD_{600nm} ~ 0,3. Detection was performed using monoclonal anti-$FasR_{MT}$ antibodies (lower panel) and anti-RpoB as loading control (upper panel).

Lipid profile in negative ion mode: signal %				
	*MtbH*37Rv	*MtbΔfasR*	*MtbΔfasR-cfasR*	*p-value**
CL	36.37 ± 2.43	20.87 ± 3.34	31.52 ± 0.78	0.0234
PE	1.54 ± 1.43	2.31 ± 1.25	3.92 ± 1.23	0.624
PG	0.74 ± 0.11	0.82 ± 0.07	0.86 ± 0.25	0.4725
PI	16.71 ± 2.26	7.49 ± 0.09	16.45 ± 3.35	0.0234
PIMs	8.97 ± 2.43	6.01 ± 1.06	9.35 ± 2.74	0.1851429
MA alpha	7.54 ± 1.43	18.44 ± 0.61	6.7 ± 2.2	0.018
MA keto	1.54 ± 0.22	2.98 ± 0.42	1.5 ± 0.62	0.0234
MA methoxy	9.66 ± 2.3	16.78 ± 0.22	13 ± 8.58	0.03825
SL	0.89 ± 0.71	0.04 ± 0.03	0.16 ± 0.04	0.0234

* T-test *MtbΔfasR vs MtbH*37Rv. Benjamini-Hochberg adjusted *p-value*.

Lipid profile in positive ion mode: signal %				
	*MtbH*37Rv	*MtbΔfasR*	*MtbΔfasR-cfasR*	*p-value**
DAG	5.91 ± 1.37	1.84 ± 1.28	3.29 ± 0.19	0.0538
TAGs	67.15 ± 2.5	76.39 ± 0.61	72.78 ± 4.64	0.0246
MMDAG	2.43 ± 0.19	3.54 ± 0.25	2.57 ± 0.12	0.0246
GroMM	0.27 ± 0.13	0.03 ± 0.01	0.14 ± 0.047	0.0898
PDIM	23.1 ± 1.56	16.94 ± 0.24	20.22 ± 4.39	0.0246
TMM	0.02 ± 0.004	0.005 ± 0.001	0.1 ± 0.01	0.0246

* T-test *MtbΔfasR vs MtbH*37Rv. Benjamini-Hochberg adjusted *p-value*.

Figure S2. Relative abundance of lipid species. The tables show the relative abundance as the total signal percentage of the different lipid classes. CL, cardiolipin; PE, phosphatidylethanolamine; PG, phosphatidylglycerol; PI, phosphatidylinositol; PIMs, phosphatidylinositol mannosides; SL, sulfoglycolipids; MA, mycolic acids; DAG, diacylglycerol; TAGs, triacylglycerol; MMDAG, monomycolyl-DAG; GroMM, glycerol monomycolate; PDIM, phthiocerol dimycocerosates; TMM, trehalose monomycolate. Results are the mean of three independent experiments ± SD (n = 3). p values were calculated with unpaired *t* test with Benjamini-Hochberg's correction between *Mtb*Δ*fasR* and *Mtb*H37Rv.

A)

Lipid profile in negative ion mode: C – chain length				
	MtbH37Rv	*MtbΔfasR*	*MtbΔfasR-cfasR*	*p-value**
CL	68.71 ±0.02	68.65 ±0.14	68.95 ±0.05	0.017
PE	34.4 ±0.28	34.32 ±0.08	34.33 ±0.21	0.909
PG	34.2 ±0.11	34.17 ±0.17	34.3 ±0.03	0.219
PI	34.93 ±0.04	34.96 ±0.12	34.99 ±0.04	0.711
MA alpha	78.64 ±0.004	78.37 ±0.04	78.52 ±0.09	0.078
MA keto	84.64 ±0.03	83.65 ±0.28	84.3 ±0.08	0.016
MA methoxy	85.34 ±0.34	84.77 ±0.02	85.12 ±0.05	0.002
SL	151.04 ±0.27	144.01 ±1.61	148.84 ±0.72	0.013

Lipid profile in positive ion mode: C – chain length				
	MtbH37Rv	*MtbΔfasR*	*MtbΔfasR-cfasR*	*p-value**
DAG	35.49 ±0.09	36.82 ±0.96	35.28 ±0.06	0.031
GroMM	47.61 ±0.15	46.67 ±0.9	46.49 ±0.93	0.018
TAGs	55.87 ±0.21	58.65 ±0.43	56.34 ±0.33	0.003
TMM alpha	82.16 ±0.13	81.22 ±0.13	80.89 ±0.4	0.237
TMM keto	87.07 ±0.13	86.07 ±0.65	86.69 ±0.15	0.106
TMM methoxy	87.51 ±0.12	86.39 ±0.31	87.32 ±0.47	0.052
PDIM A	93.19 ±0.26	91.17 ±0.21	92.65 ±0.03	0.0005

* T-test *MtbΔfasR vs MtbH*37Rv. Benjamini-Hochberg adjusted *p-value*.

B)

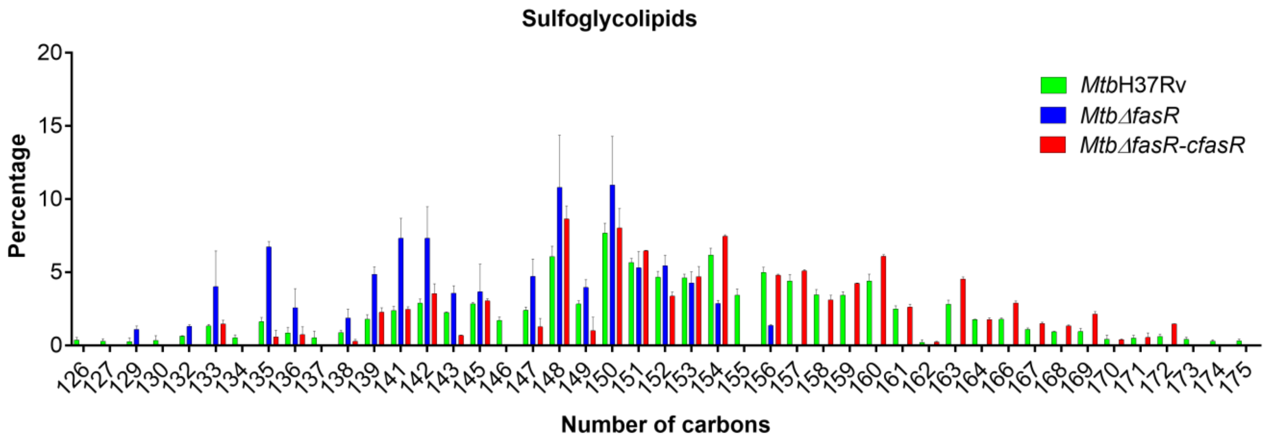

C)

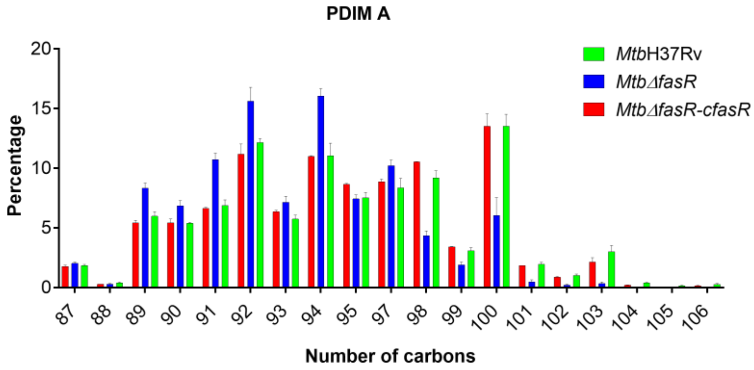

Figure S3. Detailed analysis of lipid species. (A) The tables show the weighted average of total number of carbons atoms per molecule within the population of mycolic acids (MA), phospholipids (PE, phosphatidylethanolamine; CL, cardiolipin; PG, phosphatidylglycerol; PI, phosphatidylinositol); sulfoglycolipids (SL); DAG, diacylglycerol; GroMM, glycerol monomycolate; TAGs, triacylglycerol; TMM trehalose monomycolate and phthiocerol dimycocerosates (PDIM A). The histograms show the distribution of the number of carbon atoms of SL **(B)** and PDIM **(C)** present in the wild type strain (*Mtb*H37Rv), the *fasR* complemented strain (*Mtb*Δ*fasR*-c*fasR*) and *fasR* mutant strain (*Mtb*Δ*fasR*). Results are the mean of three independent experiments ± SD (n = 3). p values were calculated with unpaired *t* test with Benjamini-Hochberg's correction between *Mtb*Δ*fasR* and *Mtb*H37Rv.

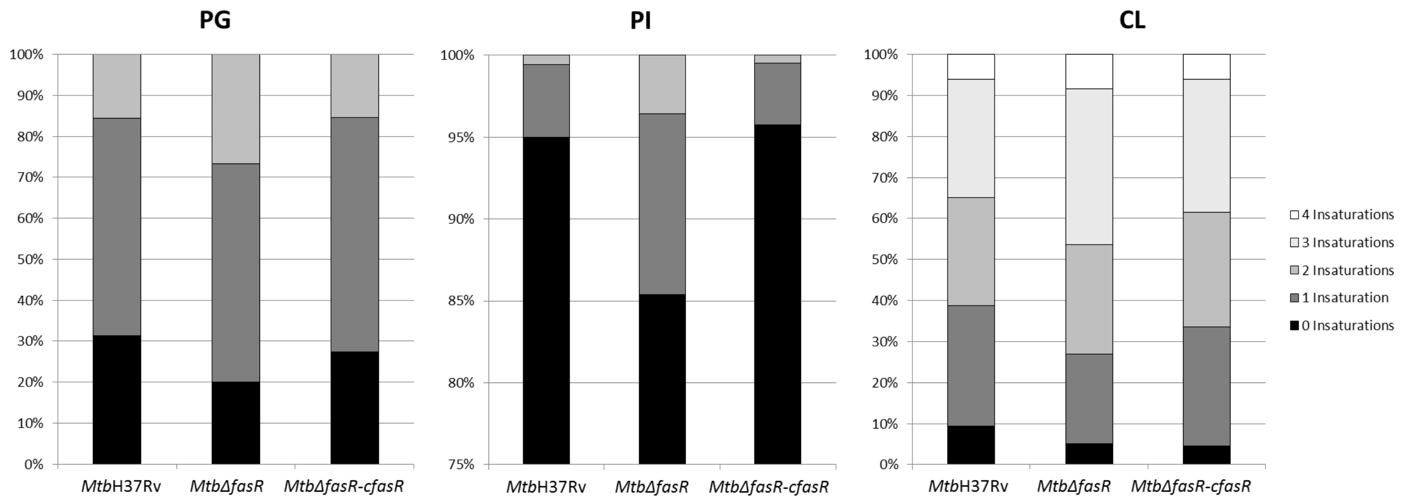

Figure S4. Saturation level of the fatty acid substituents of the phospholipids. Histograms represent the levels of unsaturation of the fatty acids constituents of cardiolipin (CL), phosphatidylglycerol (PG) and phosphatidylinositol (PI). Results are the means of three independent experiments.

References

Cole, S. T., Brosch, R., Parkhill, J., Garnier, T., Churcher, C., Harris, D., et al. (1998). Deciphering the biology of Mycobacterium tuberculosis from the complete genome sequence. *Nature* 393, 537–544.

Gomez, J. E., and Bishai, W. R. (2000). whmD is an essential mycobacterial gene required for proper septation and cell division. *Proc. Natl. Acad. Sci. U. S. A.* 97, 8554–8559. doi:10.1073/pnas.140225297.

Hanahan, D. (1983). Studies on transformation of Escherichia coli with plasmids. *J.Mol.Biol.* 166, 557–580.

Kolly, G. S., Boldrin, F., Sala, C., Dhar, N., Hartkoorn, R. C., Ventura, M., et al. (2014). Assessing the essentiality of the decaprenyl-phospho-d-arabinofuranose pathway in Mycobacterium tuberculosis using conditional mutants. *Mol. Microbiol.* 92, 194–211. doi:10.1111/mmi.12546.

Mondino, S., Gago, G., and Gramajo, H. (2013). Transcriptional regulation of fatty acid biosynthesis in mycobacteria. *Mol. Microbiol.* 89, 372–387.
